# Schistosomiasis Knowledge, Attitudes, and Practices Among School‐Going Children Aged 5–14 Years in Nelson Mandela Bay (NMB), South Africa

**DOI:** 10.1155/japr/6617259

**Published:** 2026-05-06

**Authors:** Maryline Vere, Wilma ten Ham-Baloyi, Lucy Ochola, Opeoluwa Oyedele, Takafira Mduluza, Paula Melariri

**Affiliations:** ^1^ Department of Environmental Health, Faculty of Health Sciences, Nelson Mandela University, University Way, Gqeberha, South Africa, mandela.ac.za; ^2^ Department of Nursing Science, Faculty of Health Sciences, Nelson Mandela University, University Way, Gqeberha, South Africa, mandela.ac.za; ^3^ Department of Tropical and Infectious Diseases, Institute of Primate Research (IPR), Nairobi, Kenya, primateresearch.org; ^4^ Department of Computing, Mathematical and Statistical Sciences, School of Science, University of Namibia, Windhoek, Namibia, unam.edu.na; ^5^ Department of Biochemistry and Biotechnology, University of Zimbabwe, Harare, Zimbabwe, uz.ac.zw

**Keywords:** factors, knowledge attitudes and practices, schistosomiasis, swimming

## Abstract

**Background:**

Schistosomiasis, a parasitic waterborne infection, remains a major public health challenge in disadvantaged regions, with schoolchildren (5–14 years) at high risk due to frequent water exposure. The study is aimed at assessing the knowledge, attitudes, and practices (KAP) related to schistosomiasis among school‐aged children in Nelson Mandela Bay (NMB) and examining how sociodemographic and environmental factors influence KAP outcomes.

**Methods:**

A quantitative, descriptive, cross‐sectional study was conducted among 759 schoolchildren aged 5–14 years, enrolled in Grades 0–7. Data were collected using a structured, closed‐ended, interview‐administered questionnaire, which included sections on sociodemographic characteristics, clinical history, and KAP related to schistosomiasis. Bivariate and multivariate analyses were performed to evaluate associations and describe the data using R software (Version 4.3.1).

**Results:**

Only 11% participants were aware of schistosomiasis, mainly learning from school (62%) or home (35%). Key environmental factors included urinating in rivers (44%), living near water bodies (21.1%), and swimming (11.3%). Knowledge and attitude scores showed a moderate positive correlation (*r* = 0.33; *p* < 0.001). Gender and grade level significantly influenced KAP scores, with males and older children (Grades 4–7) exhibiting better knowledge (*p* = 0.015), attitudes (*p* = 0.023), and practices (*p* = 0.001). Females had lower knowledge scores (*β* = −0.15; *p* = 0.018), while older children displayed fewer positive attitudes (*β* = 0.07; *p* = 0.038) and poorer practices (*β* = 0.11; *p* = 0.001).

**Conclusion:**

Significant gaps in knowledge, poor attitudes, and inadequate hygiene practices highlight the need for targeted education and community‐based strategies to improve KAP and reduce schistosomiasis risk in NMB schoolchildren.

## 1. Introduction

Schistosomiasis, or bilharzia, is a parasitic infection caused by worms of the *Schistosoma* genus, which are transmitted by freshwater snails [1]. Schistosomiasis poses an infection risk to more than 800 million individuals across 76 countries worldwide, with Africa being home to 46 of these countries [2, 3]. Approximately 240 million individuals are infected with schistosomiasis globally, with 85% of these infections in sub‐Saharan Africa [4]. According to de Boni et al. [5], most information on schistosomiasis in South Africa comes from KwaZulu‐Natal, followed by Limpopo and the Eastern Cape, which historically were regions with a greater burden of the disease. Annually, around 5.2 million people in South Africa need preventive therapy for schistosomiasis, with more than 90% of infections occurring in school‐aged children [6]. *Schistosoma* (*S.*) *haematobium*, the most prevalent species in South Africa, has the highest incidence of infection among males aged 5–19 years.

The genus *Schistosoma* comprises six species that have significant pathological implications for humans: *S. haematobium*, *S. mansoni*, *S. japonicum*, *S. mekongi*, *S. intercalatum*, and *S. guineensis* [7]. Approximately 280,000 deaths occur each year due to infection with the two most abundant strains of the parasite, namely, *S. haematobium* (urogenital schistosomiasis) and *S. mansoni* (intestinal schistosomiasis) [8]. Infections in humans and mollusks occur upon contact with freshwater bodies that have been contaminated with the feces of individuals infected with schistosomes. The presence of the *Bulinus* and *Biomphalaria* intermediate host snail species for *S. haematobium* and *S. mansoni* parasites, respectively, in freshwater bodies significantly influences the life cycle of the parasite and perpetuates the transmission of the disease [9]. Snail intermediate hosts for schistosomiasis thrive in slow‐moving aquatic environments like lakes and rivers due to their ability to move and eat while using less energy [10].

Transmission is especially prevalent among those living in rural areas due to inadequate sanitation and hygiene, cultural and religious practices, agriculture, recreational activities, and poor knowledge, attitudes, and practices (KAP) that facilitate disease transmission in affected areas [11, 12]. However, because of human migration, the transmission of the disease has also extended to periurban and urban areas [13]. The risk of infection is associated with demographic characteristics, such as age, gender, and occupation, as well as environmental factors [12]. In regions with a high prevalence of infection, children are exposed to contaminated slow‐moving water bodies at a younger age when they embark on swimming, fishing, farming, and household chores [14]. Moreover, preschool‐aged children below the age of 6, who accompany their guardians and carers during outdoor activities like doing laundry at the river, collecting water for household use, and bathing in the river, are at a higher risk of infection [15].

A study was conducted by Hambury [16] to determine the KAP toward schistosomiasis among school‐aged children (9–16 years old) in Grades 4–7 in KwaNobuhle, Nelson Mandela Bay (NMB). Knowledge has a significant impact on the attitudes of children and informs the practices toward factors associated with schistosomiasis transmission [17, 18]. The study found that the participants had a generally low level of knowledge and negative attitudes toward schistosomiasis. In addition, the study revealed some environmental characteristics linked to the transmission of schistosomiasis. These factors include the presence of sanitary facilities, crossing rivers along the way to school, and living near stagnant and slow‐moving water bodies that are visited regularly by the participants [19]. The aforementioned study focused on participants in Grades 4–7, aged 9–16, residing in one of the towns in NMB. The absence of data regarding the prevailing factors associated with schistosomiasis transmission in areas with possible transmission sites poses a challenge in formulating effective and targeted control strategies. The main objective of this study was to determine the factors and KAP associated with schistosomiasis transmission in school‐going children aged between 5 and 14 years in NMB, Eastern Cape Province, South Africa.

## 2. Materials and Methods

### 2.1. Study Area

**Figure 1:**
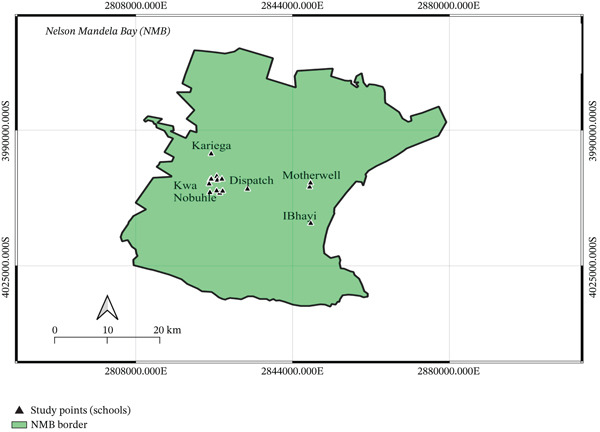
Map showing study area and data collection points (source: Map was generated by researcher using QGIS 3.34 Prizren).

The study was carried out in periurban Motherwell, Despatch, KwaNobuhle, Kariega, and Ibhayi, which are in the NMB, in the Eastern Cape Region of South Africa (refer to Figure 1 for the specific study area and data collection locations). The NMB municipality covers a total surface area of approximately 1959 km^2^, located at geographical coordinates 33°57 ^′^S, 25°36 ^′^E. In 2022, the population of NMB stood at 1.33 million people [20]. The 0–9 age group accounts for 20% of the total population, closely followed by the 10–19 age group, which represents 18% of the population [20].

The climate in NMB is marked by cool winters (May–September), warm summers (December–March), and generally dry, clear, and windy conditions year‐round [21]. During the study period (August 2023 to February 2024), the mean temperature was 22.7°C. The highest temperature (35.5°C) was recorded in April 2023 and the lowest (12°C) in July. Average annual rainfall is approximately 331 mm, with April being the wettest month (50 mm) and August the driest (3.4 mm) [22]. Environmental conditions in NMB support schistosomiasis transmission. Rivers such as the Swartkops, Papkuils, and Chatty, along with streams, ponds, and drainage channels, serve as habitats for intermediate host snails. Limited access to piped water drives communities to use these water bodies for bathing, washing, and recreation. Fishing and small‐scale irrigation farming along riverbanks also increase water contact, especially among children and farm workers, facilitating exposure to *Schistosoma* parasites and sustaining transmission.

### 2.2. Ethics

The Research Ethics Committee: Human (REC‐H) of Nelson Mandela University (H23‐HEA‐ENV‐001) (Annexure K) granted institutional ethical approval for the study prior to data collection. Additionally, authorizations and permissions from gatekeepers were acquired from the Eastern Cape Department of Education (ECDOE), the school governing boards (SGBs), and the principals of 15 primary schools who participated in the study. Prior to commencing the enrolment process, appropriate written and verbal agreement and consent were obtained from the school children and their parents or legal guardians, as they were below the age of 18.

### 2.3. Study Design

This study utilized a quantitative descriptive cross‐sectional design to investigate factors associated with schistosomiasis transmission, as well as the KAP of schoolchildren in Grades 1–7 regarding the disease. The study targeted primary schools in KwaNobuhle, Ibhayi, Kariega, Motherwell, and Despatch, collecting data from August 2023 to February 2024 (interviews were scheduled on single days per school for logistical feasibility).

### 2.4. Study Population and Sampling Strategy

A random sample of school‐going children was drawn from 15 primary schools across purposively selected five towns in NMB. The selection of towns was based on the proximity of residents to freshwater bodies, such as rivers, lakes, or ponds. The selected towns have high population densities, which increases the risk of schistosomiasis transmission due to unhygienic practices. KwaNobuhle was specifically targeted due to higher historical prevalence [23]. The study population from the 15 schools consisted of school‐going children in Grades 0–7, ranging from 5 to 14 years old. School‐going children were eligible to participate in the study if they met the following inclusion criteria: (i) They were enrolled in one of the selected schools, (ii) their age ranged from 5 to 14 years, (iii) they had written assent and consent from their parents/guardians, (iv) they could communicate in IsiXhosa and/or Afrikaans (widely spoken languages in the study area) and/or English, and (v) they were present on the day of the interview.

The South African public primary education system is typically divided into two distinct phases: lower primary education (foundation phase), which includes Grades R (0) to 3, and upper primary education (intermediate phase), which includes Grades 4–7. Consequently, all primary schools in the study area that fell within these grade ranges were eligible for inclusion and could be selected for the study. The study participants were chosen through a stratified cluster‐sampling technique, in which the entire population from each school was divided into two phases. Classes were selected randomly from each grade in each school. This method allowed for the incorporation of a predetermined number of children from every grade into the research. This ensured that each child had an equal opportunity to be selected from the population for inclusion in the study. This method ensured that the sample proportions for gender and grade were identical to the corresponding population proportions. Only participants who received parental consent were ultimately included in the study.

### 2.5. Sample Size Determination

The 2019 Eastern Cape Report on Communicable Diseases Control Directorate (CDCD) presented findings from a cross‐sectional study carried out in 2017 in the Eastern Cape Province. The overall prevalence of *S. haematobium* throughout all districts was 2%, while the prevalence of *S. mansoni* was 0.5%. The incidence of *S. haematobium* infections in NMB was recorded at 2% [23]. Using this 2% prevalence, this formula, adapted from Pourhoseingholi et al. [24], was used to calculate the appropriate sample size:

n=Z2×p×1−pd2,

where *n* is the sample size, *Z* = 1.96 represents a confidence level of 95%, *p* is the prevalence of schistosomiasis, and *d* = 1*%* is the precision error.

That is

n=1.962×0.02×10.02−0.012≅753.

Adjusting for a 10% nonresponse rate of 753 × 0.1 = 75 participants yields a minimum sample size of 753 and a maximum of 828 participants that were considered for enrolment in the study.

### 2.6. Recruitment and Enrolment Processes

**Figure 2:**
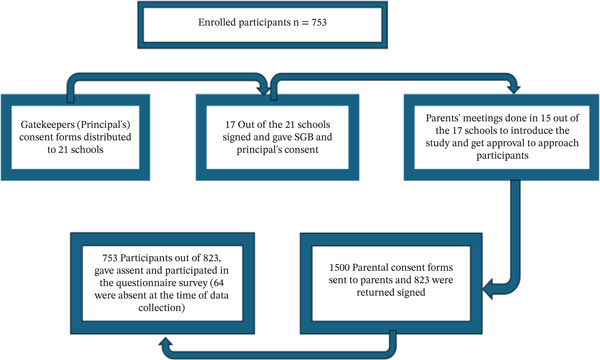
Schematic diagram of the recruitment and enrolment process.

This study was conducted to determine the KAP and factors associated with schistosomiasis transmission among school‐aged children in primary schools in NMB. Permission to access the 21 schools at the NMB was granted by the ECDOE. Consent was sought from the gatekeepers/principals of each school to ask for the school′s participation in the project. However, 17 schools consented to join, as the remaining four were denied approval by their respective school governing bodies. Meetings were scheduled with parents at the 17 schools on different days to further introduce and explain the research. Nevertheless, in two of the schools, the endeavors to engage with parents were found to be ineffective. Upon obtaining consent from the parents, we proceeded to distribute 1500 consent forms to the participants. The purpose of these forms was for the participants to take them home and have their parents or guardians sign them. A total of 823 participants submitted signed consent forms. The researcher further explained the study to the school‐going children with consent. Those who agreed and assented to participate were given forms to sign as well. During the data collection visits, a total of 64 children who had submitted signed consent were absent and, therefore, were not interviewed. As a result, they were excluded from the study. The study sample consisted of 759 participants who underwent interviews, as summarized in Figure 2.

### 2.7. Data Collection and Questionnaire Development

Data collection was conducted through face‐to‐face interviews by two trained field workers using a structured questionnaire with 48 closed‐ended, multiple‐choice questions. Each interview lasted approximately 15–20 min. The questionnaire was titled “Factors and knowledge, attitudes, and practices regarding schistosomiasis among school‐age children in NMB” and was developed after reviewing existing literature [25, 26].

The questionnaire was divided into two sections as shown in Table 1. Section A focused on sociodemographic information, clinical symptoms, and factors associated with schistosomiasis transmission. Questions in this section addressed activities such as swimming, bathing, crossing rivers without footwear, collecting water from nearby slow‐moving water bodies, and sanitation practices, as well as the presence of toilets and sources of household water. Section B aimed at assessing the children′s KAP regarding schistosomiasis, including general awareness, knowledge about snails, causes, routes of transmission, prevention, and treatment of schistosomiasis. Section B explored their sources of knowledge, whether schistosomiasis can be prevented, and whether certain practices like wearing shoes, using clean water, and seeking medical treatment are important to reduce the risk of infection. Mixed‐format questioning was used in the current survey, with a combination of multiple‐choice questions and simple dichotomous “Yes” or “No” or uncertain “I don′t know” response options [26].

**Table 1:** Components of the questionnaire.

Section	Subsection	Questions	Type of questions
Section A	Sociodemographic	Age, gender, grade, name of community, and religious affiliation	Demographic questions, closed‐ended
	Clinical variables	Infected/not infected, history of infection, and clinical symptoms	Closed‐ended, multiple choice
	Factors for schistosomiasis transmission	Swimming, bathing, crossing rivers barefoot, collection of water for domestic uses, recreational activities (swimming and fishing), water sources, sanitation, presence of toilets, and identification of snails and whether they are harmful	Closed‐ended
Section B	Knowledge	Schistosomiasis signs and symptoms, cause, mode of transmission, prevention and treatment, and sources of knowledge	Closed‐ended
	Attitude	If it is a serious disease, does it have a cure? Can it be prevented? Feces and urine as sources of infection	Closed‐ended
	Practices	Wearing shoes when going outside, washing clothes, bathing in open water sources and freshwater bodies, seeking treatment, using toilets, and urinating/defecating in rivers	Closed‐ended

The questionnaire was developed in English and translated into Afrikaans and IsiXhosa, the two most widely spoken local languages in NMB, to ensure younger children could understand the questions. A back translation into English was conducted to verify and validate the accuracy of the translations. The interviews were carried out in private rooms at the schools during break and lunch times to minimize disruption to the participants′ learning.

### 2.8. Data Analysis

Data from the survey responses of each participant were captured in the QuestionPro (https://www.questionpro.com/) data management system. Upon completion of data entry in the QuestionPro software, an Excel spreadsheet and a code sheet were generated.

The data collected from the questionnaire was processed and analyzed using Microsoft Office 365 (2019 edition). The researcher conducted data cleaning and processing, which involved carefully examining the data for any errors in the scores assigned to the risk variables related to transmission and KAP about schistosomiasis items in the questionnaire. A statistician was consulted regarding data analysis using the R software (Version 4.3.1). The scores for the KAP aspects were calculated by combining the participants′ answers to the KAP questions. A score of one was allocated for each “Yes” response, while a score of two was awarded for “No” and three for “do not know” or erroneous answers. The inclusion of the “I don′t know” option was intended to prevent conjecture [26]. “N/A” responses were retained as a separate category in analysis rather than being treated as missing data, as they represent meaningful responses. The data was analyzed and described using descriptive and inferential statistics. Various tests, such as Pearson′s correlations, independent Student′s *t*‐test, analysis of variance (ANOVA), and linear regression analysis, were employed. A multivariate regression analysis was conducted to determine the factors that are associated with schistosomiasis transmission and KAP regarding schistosomiasis. A *p* value less than 0.05 (*p* value < 0.1) was deemed statistically significant.

## 3. Results

### 3.1. Sociodemographic Characteristics

The study recorded a response rate of 92.22%. The response rate is significant, indicating the engagement and interest of participants, parents, and gatekeepers. The study involved a total of 759 participants, with 440 (58%) being males and 319 (42%) being females. The participants were primary school‐going children aged 5–14 years from KwaNobuhle, Motherwell, Kariega, Despatch, and Ibhayi. Most of the participants fell between the age category of 9 and 14 (89.9%) years, with fewer participants in the 5–8 (10.1%) year age category. Participants enrolled in the study were in Grades 0–7, with most participants in Grades 4–7 (61.5%) and a few participants in Grades 0–3 (38.5%).

Participants had different religious affiliations ranging from African Traditional Religion, mainline churches, Apostolic, Pentecostal, Muslim, and others, with a descending distribution of 454 (59.8%), 136 (17.9%), 85 (11.2%), 30 (4%), 3 (0.4%), and 51 (6.7%), respectively. The number of people in a family ranged from 1 to 3, 62 (8.2%); 4 to 6, 344 (45.3); and more than 7, 353 (46.5%). Among the total participants, 11 individuals reported traveling to and returning from rural areas during the holiday period, while the remaining 748 participants remained within NMB. Most of the participants were recruited from schools in KwaNobuhle 439 (57.8%), followed by Motherwell 152 (20%), Despatch 60 (7.9%), and both Ibhayi and Kariega, with 54 (7.1%) participants each (Table 2).

**Table 2:** Sociodemographic characteristics of study participants (*N* = 759).

Sociodemographic variable	Number of participants (*n*)	Percentage (%)
	759	100.0
Age		
5–8 years	77	10.1
9–14 years	682	89.9
Grade		
0–3	292	38.5
4–7	467	61.5
Gender		
Male	440	58.0
Female	319	42.0
Religious affiliation		
Mainline	136	17.9
Pentecostal	30	4.0
Apostolic	85	11.2
African tradition	454	59.8
Muslim	3	0.4
Other	51	6.7
How many are you in your family?		
1–3	62	8.2
4–6	344	45.3
More than 7	353	46.5
Do you travel outside Nelson Mandela Bay during the holidays, or do you stay within the city?		
Yes	11	1.8
No	720	94.5
N/A	28	3.7
If yes to the question above, from where?		
N/A	748	98.6
Rural areas	11	1.4
Town		
KwaNobuhle	439	57.8
Kariega	54	7.1
Despatch	60	7.9
Ibhayi	54	7.1
Motherwell	152	20

### 3.2. Clinical Variables and Symptoms

Of the 759 participants, relative to the responses of the questionnaire survey, a total of 4 (0.5%) had schistosomiasis infections. Specifically, 1 (0.1%) participant reported being infected with schistosomiasis during the time of the visit, while 3 (0.4%) had a history of schistosomiasis infection. Of the 759 participants, 51 (6.7%) had cough, 52 (6.9%) had diarrhea, 5 (0.7%) had muscle aches and pains, 59 (7.8%) had tummy pain, 4 (0.5%) had an itchy red blotchy and raised rash, 9 (1.2%) reported seeing blood in urine, and 627 (82.6%) participants did not report to have any of these symptoms at the time of the visit. Of the participants who reported having any of the clinical symptoms, 27 (3.7%) reported that they did nothing to treat the symptoms, 17 (2.2%) reported that they used home remedies, 6 (0.8%) reported that they went to the hospital, and 130 (17.1%) did other things to get better.

### 3.3. Availability of Hygiene Facilities

Out of the 759 participants, 698 (92%) indicated that they obtain water from municipal water sources (municipal water sources) in their homes, while 28 (3.7%) reported having household boreholes. A total of 33 (4.3%) individuals reported that they obtain their water from the community boreholes. In the current study, 160 (21.1%) stated that they resided near a river, whereas 599 (78.9%) did not. Regarding the presence of toilets in their homes, most of the participants, specifically 607 (80%) individuals, reported having functional toilets at home, whereas 152 individuals (20%) reported not having functional toilets. Among the participants who did not have functioning toilets, 39 (5.1%) resorted to using the outdoors, 22 (2.9%) used their neighbor′s toilet, and 2 (0.3%) used public toilets.

### 3.4. Factors Associated With Schistosomiasis Transmission

Among the 759 participants in the present study, the majority, 663 (87.1%), reported choosing to spend their leisure time at home, either after school or on weekends. A small fraction of participants, 3 (0.4%), engaged in fishing in the rivers nearby, whereas a considerably larger proportion, 86 (11.3%), mentioned participating in swimming activities in those same rivers. Out of the total, only 7 (0.9%) participants were involved in other activities. Among the 86 students who engage in swimming after school, 38 (44%) of them have acknowledged urinating in the water during their leisure activity. Despite these practices, all participants indicated that they engage in bathing activities at their place of residence, utilizing running tap water. Regarding the subject of crossing a river on the way to school, it was found that out of the total participants, 81 (9.1%) participants out of the total sample size reported crossing a river. Among the participants who go through a river on their way to school, it was found that half of them reported crossing barefoot, whereas the other half mentioned that they occasionally wore shoes when crossing the water body.

### 3.5. KAP Regarding Schistosomiasis

In this study of 759 participants, overall awareness and understanding of schistosomiasis were very low. Only 81 (11%) participants had heard of the disease, with information primarily obtained from school or parents, despite schistosomiasis not being part of the formal school curriculum in South Africa (Integrated School Health Programme, 2024). Among those exposed to school education, 51 (62%) recalled receiving information about the disease, while 28 (35%) learned about it at home. Knowledge of preventive measures was minimal, with 741 (97.6%) unaware of how to prevent infection and only 69 (9.1%) recognizing the presence of snail intermediate hosts in rivers, of whom just 36 (4.7%) perceived snails as harmful and 17 (2.2%) acknowledged their role in disease transmission. Misconceptions were common: A small proportion correctly identified transmission routes and preventive practices, such as avoiding contaminated water.

Attitudes toward schistosomiasis reflected limited concern and awareness. Only 65 (8.6%) considered the disease serious, 5 (0.7%) believed a cure exists, and most participants were unsure about its severity or the impact on daily life. Concern about contracting schistosomiasis was negligible, with only one participant expressing worry, while 665 (87.6%) were uncertain.

Practices potentially increasing disease risk were noted. A total of 173 (22.8%) did not consistently wear shoes outdoors, and 92 (12%) reported visiting rivers or water bodies after school, some daily. Only 21 (2.8%) actively avoided rivers to prevent infection. The low level of protective behaviors corresponds with limited knowledge and concern about the disease.

### 3.6. Differences in KAP and Demographics

The results obtained from assessing the differences in KAP scores across the participants′ demographics are presented in Table 3. The ANOVA and *t*‐tests were conducted to investigate potential relationships between demographic variables (age, gender, grade, and religious affiliation) and KAP scores. The sample mean scores were compared across different groups to identify any significant differences (Table 3).

**Table 3:** KAP score assessment (*N* = 759).

Demographic variable	*n*	Mean	Std. dev.	ANOVA (*p* value)
Knowledge score				
Age				
5–8 years	77	11.00	25.26	0.105
9–14 years	682	97.43	207.16	
Gender				
Male	440	62.86	128.55	0.015 ^∗^
Female	319	45.57	104.07	
Grade				
0–3	292	41.71	93.59	0.045 ^∗^
4–7	467	66.71	138.86	
Religious				
Mainline	136	19.43	43.86	0.141
Pentecostal	30	4.29	10.05	
Apostolic	85	12.14	26.49	
African tradition	454	64.86	133.48	
Muslim	3	0.43	0.79	
Other	51	7.29	17.96	
Attitude score				
Age				
5–8 years	77	19.25	34.57	0.318
9–14 years	682	170.50	292.42	
Gender				
Male	440	110.00	186.68	0.490
Female	319	79.75	140.36	
Grade				
0–3	292	73.00	130.92	0.023 ^∗^
4–7	467	116.75	196.07	
Religious				
Mainline	136	34.00	63.39	0.086 ^∗∗^
Pentecostal	30	7.50	13.08	
Apostolic	85	21.25	35.89	
African tradition	454	113.50	190.96	
Muslim	3	0.75	0.96	
Other	51	12.75	22.87	
Practice score				
Age				
5–8 years	77	25.67	33.83	0.182
9–14 years	682	227.33	266.84	
Gender				
Male	440	146.67	168.36	0.142
Female	319	106.33	132.43	
Grade				
0–3	292	97.33	129.26	0.001 ^∗^
4–7	467	155.67	172.34	
Religious				
Mainline	136	45.33	55.58	0.438
Pentecostal	30	10.00	11.79	
Apostolic	85	28.33	33.29	
African tradition	454	151.33	181.80	
Muslim	3	1.00	1.00	
Other	51	17.00	17.52	

^∗^Statistical significance at a 5% level.

^∗∗^Marginal significance at a 10% level.

Among the participants, a statistically significant difference was observed in the knowledge scores based on their gender (*p* value = 0.015). Males tend to have higher values (*M* = 128.55) compared to females (*M* = 104.07) and grade (*p* value = 0.045).

In terms of attitude scores, the results of the current study revealed a statistically significant difference between the two grade groups (0–3 and 4–7) (*p* = 0.023). Specifically, participants in the higher grade group exhibited higher average scores (*M* = 138.86) compared to those in the lower grade (*M* = 93.59). There was a notable difference in practice scores between students in Grades 0–3 and Grades 4–7. On average, students in higher grades achieved higher scores (*p* value = 0.001).

Interestingly, in the current study, while knowledge and practice scores did not significantly differ based on religious affiliations, attitude scores did show significant differences (*p* value = 0.086). This suggests that religious beliefs might influence participants′ attitudes toward schistosomiasis prevention, possibly due to varying cultural perspectives on health and hygiene.

### 3.7. Differences in KAP and Factors

The results obtained from assessing the differences in the scores for KAP across the participants′ risk factor characteristics are presented in Table 4. The participants who utilized municipal tap water from their homes had the highest average knowledge score (*M* = 99.71), compared to those who relied on communal borehole water (*M* = 4.71) and household borehole water (*M* = 4.00). The ANOVA indicated a marginally significant difference in knowledge means among the three groups being compared (*p* value = 0.078). These findings suggest that individuals who have access to safer and more reliable water sources, such as household municipal water sources, have higher levels of knowledge about schistosomiasis and its prevention. This is likely because households with better infrastructure have improved information dissemination and awareness regarding the disease.

**Table 4:** Comparative evaluation of factors associated with schistosomiasis infections (*N* = 759).

Risk factor	*n*	Mean	Std. dev.	ANOVA *p* value
Knowledge score				
Where do you fetch water to drink?				
Community borehole	33.00	4.71	9.57	0.078 ^∗∗^
Household borehole	28.00	4.00	9.27	
Household tap water	698.00	99.71	213.97	
Do you live near a river?				
Yes	160.00	22.86	44.14	0.530
No	599.00	85.57	188.74	
Do you have a toilet at home?				
Yes	607.00	86.71	186.73	0.006 ^∗^
No	152.00	21.71	46.08	
If no, where do you urinate or defecate?				
Bush	39.00	5.57	10.37	0.009 ^∗^
Public toilet	2.00	0.29	0.76	
Neighbor′s toilet	22.00	3.14	7.47	
Not applicable	696.00	99.43	213.96	
Do you know about snails in the river?				
Yes	69.00	9.86	18.27	0.001 ^∗^
No	690.00	98.57	214.22	
Do you cross a river on your way to school?				
Yes	69.00	9.86	21.41	0.727
No	690.00	98.57	211.01	
Where do you go to play after school or when you are not at school: Swimming?				
Yes	86.00	12.29	22.28	0.004 ^∗^
Not applicable	673.00	96.14	210.21	
Where do you go to play after school or when you are not at school: Fishing?				
Yes	3.00	0.43	0.53	0.177
Not applicable	756.00	108.00	232.13	
Where do you go to play after school or when you are not at school: At home with friends?				
Yes	714.00	102.00	221.50	0.003 ^∗^
Not applicable	45.00	6.43	10.98	
When you are swimming in the river, do you urinate in the water: Yes?				
Yes	38.00	5.43	9.20	0.001 ^∗^
Not applicable	721.00	103.00	223.28	
Attitude score				
Where do you fetch water to drink?				
Community borehole	33.00	8.25	11.44	< 0.001 ^∗^
Household borehole	28.00	7.00	8.76	
Household tap water	698.00	174.50	306.95	
Do you live near a river?				
Yes	160.00	40.00	63.67	0.016 ^∗^
No	599.00	149.75	263.38	
Do you have a toilet at home?				
Yes	607.00	151.75	263.35	0.684
No	152.00	38.00	63.70	
If no, where do you urinate or defecate?				
Bush	39.00	9.75	14.97	0.025 ^∗^
Public toilet	2.00	0.50	0.58	
Neighbor′s toilet	22.00	5.50	7.85	
Not applicable	696.00	174.00	303.96	
Do you know about snails in the river?				
Yes	69.00	17.25	19.14	< 0.001 ^∗^
No	690.00	172.50	308.21	
Do you cross a river on your way to school?				
Yes	69.00	17.25	27.93	0.117
No	690.00	172.50	299.10	
Where do you go to play after school or when you are not at school: Swimming?				
Yes	86.00	21.50	24.69	< 0.001 ^∗^
Not applicable	673.00	168.25	302.93	
Where do you go to play after school or when you are not at school: Fishing?				
Yes	3.00	0.75	0.50	0.001 ^∗^
Not applicable	756.00	189.00	326.79	
Where do you go to play after school or when you are not at school: At home with friends?				
Yes	714.00	178.50	316.30	< 0.001 ^∗^
Not applicable	45.00	11.25	11.00	
When you are swimming in the river, do you urinate in the water?				
Yes	38.00	9.50	8.58	< 0.001 ^∗^
Not applicable	721.00	180.25	320.30	
Practice score				
Where do you fetch water to drink?				
Community borehole	33.00	11.00	11.00	0.001 ^∗^
Household borehole	28.00	9.33	8.14	
Household tap water	698.00	232.67	284.81	
Do you live near a river?				
Yes	160.00	53.33	61.76	0.661
No	599.00	199.67	238.80	
Do you have a toilet at home?				
Yes	607.00	202.33	241.43	0.679
No	152.00	50.67	59.08	
If not, where do you urinate or defecate?				
Bush	39.00	13.00	15.13	0.500
Public toilet	2.00	0.67	0.58	
Neighbor′s toilet	22.00	7.33	8.08	
Not applicable	696.00	232.00	276.94	
Do you know about snails in the river?				
Yes	69.00	23.00	28.58	0.573
No	690.00	230.00	271.95	
Do you cross a river on your way to school?				
Yes	69.00	23.00	26.91	0.748
No	690.00	230.00	273.61	
Where do you go to play after school or when you are not at school: Swimming?				
Yes	86.00	28.67	35.73	0.625
Not applicable	673.00	224.33	264.89	
Where do you go to play after school or when you are not at school: Fishing?				
Yes	3.00	1.00	1.00	0.077 ^∗∗^
Not applicable	756.00	252.00	300.23	
Where do you go to play after school or when you are not at school: At home with friends?				
Yes	714.00	238.00	284.84	0.193
Not applicable	45.00	15.00	15.72	
When you are swimming in the river, do you urinate in the water?				
Yes	38.00	12.67	15.53	0.767
Not applicable	721.00	240.33	284.98	

^∗^Statistical significance at a 5% level.

^∗∗^Marginal significance at a 10% level.

### 3.8. Impact of Factors on KAP Scores

Participants with toilets at home had significantly higher knowledge scores (*M* = 86.71) than those without (*M* = 21.71; *p* = 0.006), with the highest scores observed among those reporting “Not applicable” (*M* = 99.43; *p* = 0.009). Lack of awareness about snails, an intermediate host, was associated with higher knowledge scores (*M* = 98.57 vs. 9.86; *p* = 0.001), highlighting gaps in understanding specific transmission pathways. Engagement in activities exposing participants to water bodies, such as swimming (*M* = 12.29 vs. 96.14; *p* = 0.004), playing outdoors (*M* = 102.00 vs. 6.43; *p* = 0.003), or urinating in rivers (*M* = 5.43 vs. 103.00; *p* = 0.001), was associated with lower knowledge scores, suggesting limited awareness among those at higher risk of exposure.

Attitudes toward schistosomiasis varied by environmental factors. Participants using household tap water scored highest (*M* = 174.50) compared to those using boreholes (*p* < 0.001). Living near a river corresponded with lower attitude scores (*M* = 40.00 vs. 149.75; *p* = 0.016). Access to sanitation at home correlated with more positive attitudes (*M* = 174.00 for “Not applicable”; *p* = 0.025). Paradoxically, awareness of snails′ role in transmission was associated with lower attitude scores (*M* = 17.25 vs. 172.50; *p* < 0.001), indicating that knowledge does not automatically translate into positive attitudes. Social behaviors, such as playing at home with friends, improved attitude scores (*M* = 178.50 vs. 11.25; *p* < 0.001), while risky behaviors, like urinating in rivers, were linked to lower scores (*M* = 9.50 vs. 180.25; *p* < 0.001).

Significant differences in practices were observed based on sources of drinking water (*p* = 0.001), with participants using municipal tap water reporting higher practice scores (*M* = 232.67) than those using borehole water. Fishing was associated with lower practice scores (*M* = 1.00 vs. 252.00; *p* = 0.077), suggesting that environmental exposure influences the adoption of preventive behaviors (Table 4).

### 3.9. Multivariate Regression Analysis of Demographic Factors Related to the KAP of Schistosomiasis Among Study Participants

Table 5 presents multivariate regression results examining associations between demographic factors and KAP scores on schistosomiasis among 759 participants. Females had significantly lower knowledge scores than males (*β* = −0.15; *p* = 0.018), likely due to reduced exposure to outdoor activities where educational messages are received. Children practicing African traditional religions had higher knowledge scores (*β* = 0.22; *p* = 0.010). Attitudes toward schistosomiasis prevention were less positive in Grades 4–7 compared to Grades 0–3 (*β* = −0.07; *p* = 0.038), with religious affiliation influencing attitudes—Apostolic (*β* = 0.12; *p* = 0.041) and African traditional religion followers (*β* = 0.11; *p* = 0.014) showing more positive attitudes. Practices also declined in Grades 4–7 (*β* = −0.11; *p* = 0.001), reflecting higher risk from activities such as swimming and fishing. These findings highlight the need for targeted, age‐ and gender‐sensitive health education.

**Table 5:** Multivariate regression results on the association between factors and KAP scores (*N* = 759).

Variable	Knowledge score			Attitude score			Practice score		
	Estimate	Standard error	*p*value	Estimate	Standard error	*p* value	Estimate	Standard error	*p* value
Age									
5–8 years (ref)									
9–14 years	0.17	0.11	0.141	0.02	0.06	0.721	0.01	0.05	0.818
Gender									
Male (ref)									
Female	−0.15	0.07	0.018 ^∗^	−0.02	0.03	0.56	−0.05	0.03	0.131
Grade									
0–3 (ref)									
4–7	0.09	0.07	0.179	0.07	0.03	0.038 ^∗^	0.11	0.03	0.001 ^∗^
Religious									
Mainline (ref)									
Pentecostal	0.01	0.18	0.94	0.05	0.09	0.547	0.03	0.09	0.704
Apostolic	0.18	0.12	0.145	0.12	0.06	0.041 ^∗^	0.03	0.06	0.669
African tradition	0.22	0.09	0.010 ^∗^	0.11	0.04	0.014 ^∗^	0.01	0.04	0.879
Muslim	0.14	0.51	0.788	0.27	0.26	0.289	0.11	0.25	0.664
Other	−0.03	0.15	0.836	0.05	0.07	0.466	0.12	0.07	0.101
Knowledge				0.66	0.07	< 0.001	0.11	0.07	0.11
(Intercept)	0.02	0.12	0.891	0	0.06	0.994	0.16	0.06	0.009

Abbreviation: ref, reference category.

^∗^Statistical significance at a 5% level.

^∗∗^Marginal significance at a 10% level.

Table 6 presents the results of a multivariate regression analysis examining the associations between environmental factors and KAP regarding schistosomiasis among 759 school‐going children in NMB. Children without a toilet at home demonstrated higher knowledge scores compared to those with household toilets (*β* = 0.19; *p* = 0.055), suggesting that lack of formal sanitation may expose children to greater awareness of disease risks in their environment. Attitudes toward schistosomiasis prevention were less positive among children with household tap water compared to those using community boreholes (*β* = −0.12; *p* = 0.099). This may reflect a perception that “safe” water sources reduce infection risk, which can diminish motivation for preventive behaviors. In contrast, children relying on community boreholes may perceive higher exposure risk, leading to more proactive attitudes. Children with household boreholes reported better preventive practices than those using community boreholes (*β* = 0.20; *p* = 0.066), likely due to greater control over water quality and consistent implementation of preventive measures. However, children with household tap water had slightly lower practice scores compared to community borehole users (*β* = −0.14; *p* = 0.074).

**Table 6:** Regression analysis results on the association between factors and KAP scores (*N* = 759).

Variable	Knowledge score			Attitude score			Practice score		
	Estimate	Standard error	*p*value	Estimate	Standard error	*p*value	Estimate	Standard error	*p* value
Where do you fetch water to drink?									
Community borehole (ref)									
Household borehole	−0.17	0.23	0.469	0.09	0.11	0.392	0.2	0.11	0.066 ^∗^
Household tap water	−0.18	0.16	0.257	−0.12	0.07	0.099 ^∗∗^	−0.14	0.08	0.074 ^∗^
Do you live near a river?									
Yes (ref)									
No	0.01	0.09	0.876	0.04	0.04	0.382	−0.04	0.04	0.333
Do you have a toilet at home?									
Yes (ref)									
No	0.19	0.1	0.055 ^∗^	−0.05	0.05	0.259	−0.02	0.05	0.718
If not, where do you urinate or defecate?									
Bush (ref)									
Public toilet	−0.54	0.64	0.401	0.81	0.3	0.007 ^∗^	0.06	0.31	0.836
Neighbor′s toilet	−0.55	0.23	0.020 ^∗^	0.09	0.11	0.387	−0.01	0.11	0.945
Not applicable	−0.21	0.17	0.214	−0.04	0.08	0.584	−0.07	0.08	0.415
Do you know about snails in the river?									
Yes (ref)									
No	−0.24	0.14	0.091 ^∗∗^	−0.22	0.07	0.001 ^∗^	0.09	0.07	0.216

Abbreviation: ref, reference category.

^∗^Statistical significance at a 5% level.

^∗∗^Marginal significance at a 10% level.

A positive correlation was observed between knowledge and attitudes, with each 1% increase in knowledge score associated with a 0.66‐point increase in attitude score, highlighting the critical role of awareness in shaping preventive behaviors.

These findings emphasize the complex interplay between environmental factors, perception of water safety, and schistosomiasis‐related KAP. Access to sanitation and water sources influences not only awareness but also the adoption of preventive behaviors, underlining the need for targeted health education that accounts for both environmental and behavioral contexts.

## 4. Discussion

The primary outcomes of this study were schistosomiasis‐related knowledge, attitudes, and preventive practices among school‐aged children in NMB, while secondary outcomes included the sociodemographic and environmental factors influencing these KAP measures, as well as the sources of information and the correlation between KAP. Our findings demonstrate that schistosomiasis‐related knowledge among children in NMB is significantly patterned by age, gender, education level, and environmental context. Older children and boys exhibited higher knowledge scores, while access to sanitation and water infrastructure was positively associated with attitudes toward prevention. However, and critically, increased knowledge did not consistently correspond to more positive preventive attitudes or practices. This disconnect indicates that awareness alone is insufficient to drive effective prevention. Thus, the study shows that schistosomiasis prevention among children is constrained not by lack of information per se but by contextual, structural, and perceptual barriers that limit the translation of knowledge into action.

A systematic review by Sacolo et al. [27], which analyzed 12 studies from sub‐Saharan Africa, similarly demonstrated that these variables were consistently associated with higher awareness levels. The review attributed males′ greater knowledge to occupational exposure, as health education programs often target fishermen and farmers. While Sacolo et al. [27] analysis encompassed both adults and children, the current study focused exclusively on school‐aged children, allowing for a more nuanced understanding of how awareness develops in this age group. Comparable findings were reported by Gabaake et al. [28] in Botswana, where male learners had significantly higher knowledge scores than females, further confirming gender as a key determinant of schistosomiasis awareness. These patterns were also observed by Hambury et al. [19] in KwaNobuhle, Eastern Cape, who found that older pupils and boys displayed greater understanding of schistosomiasis transmission than their younger or female counterparts. However, there are notable differences between the two studies. While Hambury et al. [19] documented similar trends among Grade 4–7 learners in a single Eastern Cape community, the present study extends these observations by including a broader age range (5–14 years), multiple communities, and multivariate modeling. This approach allowed identification of independent predictors of KAP outcomes and provided a more representative district‐level assessment of schistosomiasis awareness.

An improvement in knowledge and positive attitudes with increasing age and grade level suggests that education is a significant enabler of health awareness. Older learners may be more exposed to school‐based health topics or peer discussions about disease prevention. The link between sanitation infrastructure and higher knowledge scores observed in this study further underscores the importance of environmental and household conditions. Children from homes with functioning toilets and access to municipal water demonstrated more favorable attitudes, likely due to lower exposure risk and greater access to health information. This finding aligns with Grimes et al. and Ngasala et al. [29, 30], who emphasized that access to safe water and sanitation reduces transmission and enhances health literacy. Conversely, children living near rivers, where exposure risk is higher, showed fewer positive attitudes, possibly reflecting a perception that infection is unavoidable or an indication of limited outreach in these areas.

Although knowledge about snail vectors and transmission pathways was high, this study found that awareness alone did not translate into positive attitudes toward prevention. This gap between knowledge and practice mirrors trends observed by Hambury et al. and Sacolo et al. [19, 27], who both noted that factual knowledge of schistosomiasis often fails to prompt preventive action. The lower attitude ratings among informed participants suggest the need for educational programs that not only disseminate information but also reinforce the value of practical prevention strategies. Comprehensive health education, delivered through schools and community engagement, should focus on fostering motivation, agency, and consistent safe water practices rather than mere knowledge acquisition.

Exposure‐related activities emerged as significant predictors of attitudes. Children who reported swimming or fishing in rivers had markedly lower attitude scores compared to those who did not engage in these activities. These findings align with evidence from Kabuyaya et al. [31], who identified swimming and fishing as major factors for schistosomiasis transmission in South African endemic areas. Prolonged contact with freshwater facilitates exposure to infected cercariae, while urination in or near rivers perpetuates the transmission cycle [32]. The present results underscore the need for awareness campaigns targeting children who frequently use rivers for recreation or livelihood activities, emphasizing safer alternatives and the importance of avoiding contaminated water sources.

Interestingly, children with access to household tap water displayed fewer positive attitudes than those using public or shared facilities. This may indicate perceived safety associated with improved water sources, leading to complacency. In contrast, children relying on public toilets showed stronger preventive attitudes, possibly due to more frequent exposure to health messages within community settings. Awareness of snail presence was also associated with higher attitude scores, suggesting that understanding the environmental aspects of transmission enhances risk perception and responsiveness. Similar observations were made by Nwoko et al. [33], who reported that environmental awareness contributes to more proactive disease prevention efforts.

The study also revealed limited health‐seeking practices, with only one‐fifth of participants reporting schistosomiasis‐related symptoms to healthcare facilities. This pattern reflects findings from Danso‐Appiah et al. [34] who noted that individuals with urinary schistosomiasis are less likely to seek medical care than those with intestinal infections. Socioeconomic factors likely contribute to this pattern, as financial constraints and poor healthcare access discourage prompt medical attention [35, 36]. Poverty remains a major determinant of schistosomiasis risk in NMB, where limited sanitation, overcrowding, and unemployment exacerbate vulnerability [37, 38]. Addressing these systemic inequalities is therefore essential for improving early diagnosis and treatment.

Reported symptoms such as abdominal pain, diarrhea, and hematuria are consistent with both acute and chronic schistosomiasis, as described by Dejon‐Agobé et al. [38] and Anyolitho et al. [39]. Although parasitological testing was not conducted, the presence of these characteristic symptoms, coupled with documented water contact, strongly suggests continued local transmission. Like Hambury et al. [19], this study demonstrates that despite existing knowledge, infection risks persist due to insufficient preventive practice and suboptimal environmental management. These findings emphasize the urgent need for targeted interventions addressing both infrastructure and education in endemic urban communities.

Gender and age differences remain consistent with established epidemiological patterns. Boys, who are more likely to engage in outdoor activities, tend to have greater knowledge but also higher exposure risk [17, 40]. Girls, on the other hand, often exhibit lower knowledge levels due to reduced participation in high‐risk water activities, despite facing other domestic exposures [41]. Older children also demonstrated higher knowledge scores, corroborating studies that link age with increased understanding of disease transmission [42]. Religious practices involving frequent ablutions, as reported by Otuneme et al. [43], may further elevate exposure risk, reinforcing the need for culturally tailored health education strategies.

This study had limitations. The absence of parasitological testing and malacological surveys precluded confirmation of active infection and identification of snail intermediate hosts, limiting precision in transmission mapping. Additionally, the KAP instrument was not formally validated for internal consistency or test–retest reliability, which may affect the interpretation of attitude constructs. Self‐reported practices and symptoms are also subject to recall and social desirability bias.

Despite these constraints, the findings have important theoretical and practical implications. Theoretically, the study reinforces the distinction between knowledge acquisition and preventive action, highlighting the mediating role of environmental context and perceived efficacy. Practically, the results highlight the urgency of integrating schistosomiasis education into the formal school curriculum in South Africa, as ad hoc or incidental learning appears insufficient. Curriculum‐based instruction, combined with school‐level WASH improvements and community engagement, could foster more durable preventive attitudes.

Future studies should incorporate parasitological and malacological surveillance to identify transmission hotspots and validate risk patterns. Development and validation of a child‐appropriate schistosomiasis KAP instrument are also needed, with explicit differentiation between threat appraisal and coping appraisal. Longitudinal evaluations of school‐based interventions that combine education with tangible protective measures, such as safe water access, structured play alternatives, and routine deworming, would further clarify pathways to sustained prevention.

## 5. Conclusion

This study shows that schistosomiasis awareness among school‐aged children in NMB is uneven and does not consistently translate into prevention. Knowledge levels vary by age, gender, sanitation access, and environmental exposure, with children engaging in water‐contact activities remaining at elevated risk. By extending previous work to a broader urban context, the findings confirm that information alone is insufficient for sustained control. The absence of formal schistosomiasis education in the school curriculum, combined with persistent environmental exposure, limits effective prevention. Integrated strategies linking school‐based education, improved WASH infrastructure, and targeted interventions for high‐risk groups are therefore essential to reduce residual transmission in low‐endemic urban settings.

## Author Contributions

M.V. (PhD candidate) conceived and designed the study, conducted fieldwork, performed data collection, and wrote the manuscript. W.t.H‐B. and L.O. (co‐supervisors) provided supervision, contributed to the study design, and critically reviewed the manuscript. O.O. (statistician) performed statistical analyses and contributed to data interpretation. T.M. (consultant) served as a project consultant, offering technical guidance and expertise. P.M. (main supervisor) oversaw the entire study, provided critical insights, and reviewed the manuscript.

## Funding

This study was funded by the Water Research Commission, 10.13039/501100004424, 2022/2023‐00834.

## Disclosure

All authors read and approved the final version of the manuscript.

## Conflicts of Interest

The authors declare no conflicts of interest.

## Data Availability

The data that support the findings of this study are available on request from the corresponding author. The data are not publicly available due to privacy or ethical restrictions.
